# The mobile resistome in the water–soil–air nexus: horizontal gene transfer and environmental dissemination of antimicrobial resistance genes

**DOI:** 10.1093/femsec/fiag064

**Published:** 2026-06-17

**Authors:** Christian Joseph N Ong, Rahmatullah Nazari, Kevin Smith P Cabuhat, Jerico Bautista Ogaya, Mohamed Mustaf Ahmed, Deborah Oluwaseun Shomuyiwa, Shuaibu Saidu Musa, Oshibe Joseph Daberechi, Yusuf Hared Abdi, Rich Milton R Dulay, Don Eliseo Lucero-Prisno

**Affiliations:** Department of Biology, College of Science, De La Salle University, Manila 1004, Philippines; Faculty of Public Health, Diponegoro University, Semarang 50275, Indonesia; Department of Biology, College of Science, De La Salle University, Manila 1004, Philippines; Basic Education Department, La Consolacion University Philippines, Malolos, Bulacan 3000, Philippines; Department of Medical Technology, Institute of Health Sciences and Nursing, Far Eastern University, Manila 1008, Philippines; Faculty of Medicine and Health Sciences, SIMAD University, Mogadishu 252, Somalia; Department of Health Promotion & Behavior, College of Public Health, University of Georgia, Athens, GA 30602, United States; School of Global Health, Faculty of Medicine, Chulalongkorn University, Bangkok 10330, Thailand; Department of Nursing Science, Ahmadu Bello University, Zaria 810107, Nigeria; Department of Medical Laboratory Science, Ebonyi State University, Abakaliki 480214, Nigeria; Center for Health Research and Innovation, Somali National University, Mogadishu 252, Somalia; Faculty of Medicine and Health Science, Hormuud University, Mogadishu 252, Somalia; Department of Biology, College of Science, De La Salle University, Manila 1004, Philippines; Center for Tropical Mushroom Research and Development, Department of Biological Sciences, College of Arts and Sciences, Central Luzon State University, Science City of Muñoz, Nueva Ecija 3120, Philippines; Department of Global Health and Development, London School of Hygiene and Tropical Medicine, London WC1E 7HT, United Kingdom; Research and Innovation Office, Southern Leyte State University, Sogod, Southern Leyte 6606, Philippines; Research and Development and Community Extension, John B. Lacson Maritime University, Iloilo City 5000, Philippines

**Keywords:** mobile resistome, antimicrobial resistance genes (ARGs), environmental resistome, horizontal gene transfer, mobile genetic elements, One Health, water–soil–air nexus

## Abstract

The rapid emergence and global dissemination of antimicrobial resistance pose a serious threat to public health, environmental sustainability, and economic development. Central to this crisis is the resistome, defined as the collection of all antimicrobial resistance genes (ARGs) present in pathogenic and non-pathogenic micro-organisms across clinical, agricultural, and natural ecosystems. The environmental resistome plays a crucial role in the evolution and transmission of resistance, serving as both a reservoir and a conduit for ARG exchange through horizontal gene transfer. This review provides a comprehensive overview of the structure, diversity, and dynamics of the resistome, with emphasis on the interconnected water–soil–air continuum. Key mechanisms driving resistome dissemination, including mobile genetic elements such as plasmids, integrons, transposons, and bacteriophages, are discussed alongside the major routes of gene transfer, conjugation, transformation, and transduction. The review highlights anthropogenic drivers that intensify resistome expansion, including antibiotic misuse, wastewater discharge, agricultural runoff, and exposure to heavy metals, pesticides, and disinfectants, which promote co-selection. Advances in resistome profiling approaches, such as quantitative PCR, metagenomics, long-read sequencing, and functional metagenomics, are critically evaluated for their capacity to resolve ARG diversity, mobility, and host associations.

## Teaser

Antimicrobial resistance genes spread globally through water, soil, and air via mobile genetic elements and horizontal gene transfer, driven by human activities and detectable through advanced environmental monitoring tools.

## Introduction

Antimicrobial resistance (AMR) has become one of the most pressing threats to global public health in the twenty-first century. Historically, AMR has been examined primarily through a clinical lens; however, growing evidence indicates that resistance emergence and dissemination are strongly influenced by environmental processes operating across interconnected human, animal, and ecological systems. The World Health Organization (WHO) and the United Nations Environment Programme (UNEP) have emphasized that the environment functions not merely as a passive recipient of resistant micro-organisms but as an active reservoir and evolutionary arena where antimicrobial resistance genes (ARGs) are maintained, exchanged, and amplified (Environment UN 2023). Central to this perspective is the environmental resistome, defined as the collection of antibiotic resistance genes (ARGs) within environmental matrices (Topp et al. 2026). The magnitude of the AMR crisis is reflected in projections estimating up to 10 million annual deaths by 2050 if current trends continue (De Kraker et al. 2016). Within the One Health framework, antibiotic use in human medicine and intensive livestock production contributes to environmental contamination through wastewater, manure, and agricultural runoff, creating selective pressures that favor the persistence and dissemination of resistance determinants (Wang et al. 2023). Despite increasing recognition of these interconnected processes, discussions of AMR remain largely compartmentalized among clinical, veterinary, and environmental disciplines, often treating resistance genes, microbial communities, and ecological drivers as separate entities. This fragmentation has limited the development of an integrated understanding of the resistome as a dynamic ecological network linking environmental reservoirs to resistance emergence across One Health systems.

The environment serves as a “melting pot” where clinical pathogens and environmental bacteria interact, facilitating the exchange of genetic material under the selective pressures of residual antibiotics, heavy metals, and biocides. As a result, environmental habitats, especially soil and water, act as reservoirs for novel resistance genes and play a critical role in their global spread (Pruden et al. 2013). To truly understand AMR, we must extend our focus beyond clinical settings and investigate the ecological systems that underpin life itself (Nass and Zaher 2025). Understanding the complexities of antimicrobial resistance requires distinguishing between intrinsic resistance, acquired resistance, and bacterial persistence (Reygaert 2018). The term environmental resistome refers to the entire array of ARGs present within a specific ecological niche, which encompasses those found in non-pathogenic, indigenous soil or water bacteria (Kim and Cha 2021). This resistome is extensive and ancient, with many ARGs having evolved millions of years ago as adaptive defenses for microbes that produce natural antibiotics (Nass and Zaher 2025).

Nevertheless, not all ARGs equally threaten human health, prompting the need to explore the mobile resistome. While the environmental resistome serves as a reservoir of resistance potential, the mobile resistome comprises ARGs associated with mobile genetic elements (MGEs), such as plasmids, transposons, and integrons (Bengtsson-Palme et al 2018, Kim and Cha 2021). These elements act as the primary vehicles for horizontal gene transfer (HGT), allowing resistance to shift from harmless environmental bacteria to high-risk human pathogens. Current research underscores that merely identifying the presence of an ARG is insufficient for risk assessment. To accurately evaluate these risks, we must consider the “mobility potential” of these genes. For instance, a gene integrated into the chromosomal structure of a non-pathogenic soil microbe poses a lower immediate risk compared to that same gene located on a broad-host-range plasmid in a wastewater treatment facility (Manaia 2017). This review seeks to synthesize existing knowledge concerning the ecological dynamics of AMR across the Water-Soil-Air Nexus. Previous literature has often examined these compartments in isolation; however, this paper posits that the fluid movement of ARGs among these media is a primary factor driving the persistence of global resistance. By emphasizing these intersections, the review identifies critical “hotspots” for intervention. Ultimately, the aim is to develop a predictive framework for AMR, where environmental monitoring can function as an early-warning mechanism for emerging clinical resistance.

## The genetic drivers of ARG mobility

MGEs serve as the primary engines driving the HGT of ARGs, facilitating their rapid dissemination across bacterial populations in diverse environments (Tokuda and Shintani 2024). Plasmids, as self-replicating extrachromosomal DNA molecules, play a pivotal role due to their broad-host-range capabilities and classification into incompatibility (Inc) groups such as IncP, IncN, and IncF, which are frequently detected in environmental reservoirs like wastewater and agricultural soils (Vrancianu et al. 2020). These plasmids often carry multiple ARGs, enabling multidrug resistance, and their conjugative properties allow transfer between phylogenetically distant bacteria, exacerbating ARG mobility in anthropogenic-impacted ecosystems (Abe et al. 2020, Vrancianu et al. 2020). Transposons and insertion sequences (IS) further amplify ARG spread by mobilizing resistance cassettes onto plasmids or chromosomes through cut-and-paste or copy-out-paste-in mechanisms (Vrancianu et al 2020). Composite transposons, bracketed by IS elements like IS26 or ISCR, promote the assembly of ARG clusters, while their excision and reintegration enhance plasmid plasticity, as evidenced in clinical and environmental Enterobacteriaceae isolates (Abe et al 2020, Vrancianu et al. 2020). This intra- and intermolecular transposition is particularly concerning in high-density biofilms, where proximity fosters recombination events (Abe et al. 2020).

The mechanisms of HGT perpetuate ARG dissemination through distinct yet interconnected pathways (Abe et al. 2020). Conjugation, the predominant driver, involves direct cell-to-cell contact via type IV secretion systems, transferring conjugative or mobilizable plasmids at high frequencies (10¹ to 10⁻³ transconjugants per donor) in biofilms and gut microbiomes (Liu et al. 2023, Li et al. 2025a). Environmental factors like antibiotics and heavy metals enhance conjugative transfer by inducing relaxase and pilus expression (Liu et al. 2023). Transformation entails the uptake of free extracellular DNA (eDNA) from lysed cells, prevalent in competent Gram-positive and Gram-negative bacteria within nutrient-rich matrices like sediments (Liu et al. 2024). Competence genes (e.g. comEA, comEC) facilitate DNA binding and internalization, with ARGs persisting as stable eDNA in biofilms. Though less efficient than conjugation (10⁻⁵ to 10⁻⁸), transformation contributes cumulatively in ARG-polluted sites (Nazarian et al. 2018, Abe et al. 2020, Liu et al. 2024). Transduction, mediated by bacteriophages, transfers ARGs via generalized (packaged host DNA) or specialized (lysogenic excision) mechanisms, with the phageome acting as a reservoir for resistance elements like blaTEM and tetA (Abdulhussien et al. 2025, Li et al. 2025b). Phage bursts in wastewater amplify transduction, linking viromes to ARG evolution across bacterial genera. Collectively, these MGE-HGT dynamics underscore the need for targeted interventions to curb ARG proliferation in vulnerable ecosystems (Li et al. 2025a).

## Quantitative methodologies for tracking the mobile resistome

### Absolute quantification of ARGs and MGEs (qPCR/ddPCR)

Targeted qPCR and ddPCR remain among the most defensible approaches for absolute quantification of sentinel ARGs and mobility proxies (for example *sul1, qnr* variants, and *intI1*) when the primary objective is longitudinal trend tracking across environmental matrices and timepoints rather than exhaustive resistome discovery (Falcó et al. 2025). Compared with sequencing-based approaches, qPCR-based methods offer lower analytical cost, faster turnaround time, and greater standardization potential, making them particularly suitable for routine surveillance and regulatory monitoring programs where high sample throughput is required. As summarized in the comparison presented in Table 1, qPCR/ddPCR are particularly effective for long-term surveillance, temporal trend analysis, source tracking, and regulatory monitoring because they provide highly sensitive and quantitative measurements of predefined ARGs and MGEs across samples and timepoints. In many environmental applications, qPCR is sufficient when the research question focuses primarily on whether resistance burdens increase, decrease, or persist over time, especially for well-established marker genes linked to anthropogenic contamination or wastewater impact (Falcó et al. 2025). ddPCR further improves analytical robustness by providing calibration-free absolute quantification and greater tolerance to PCR inhibitors, which is advantageous for complex matrices such as sludge, sediments, aerosols, and organic-rich soils where standard-curve drift and matrix interference can compromise qPCR estimates (Falcó et al. 2025). However, ddPCR throughput and per-sample cost remain limiting factors for dense spatiotemporal surveillance designs, so qPCR often remains the pragmatic front-line platform, with ddPCR reserved for low-abundance targets, highly inhibited samples, or validation of critical datasets (Falcó et al. 2025)

**Table 1:** Critical comparison of quantitative methods for mobile resistome surveillance across environmental systems, highlighting analytical strengths, limitations, and unresolved inference gaps.

Method	Principal contribution	Most appropriate applications	Major limitations	Inference gaps that may require complementary approaches	Representative references
qPCR/ddPCR	Provides highly sensitive and quantitative measurements of predefined ARGs and MGEs, enabling direct estimation of gene abundance across samples and time points.	Long-term surveillance, temporal trend analysis, source tracking, regulatory monitoring, and quantification of sentinel markers such as *sul1, qnr* variants, and *intI1*.	Restricted to targeted genes; cannot detect novel ARGs; provides limited information on taxonomic hosts, genomic context, or transferability.	Elevated ARG abundance can be quantified, but whether genes are plasmid-associated, linked to MGEs, or actively exchanged among hosts generally cannot be determined without sequencing-based approaches.	(Falcó et al. 2025, Yin et al. 2025)
Shotgun metagenomics (short-read)	Enables comprehensive characterization of ARG diversity, microbial community composition, and co-occurring resistance determinants within a single dataset.	Resistome surveys, comparative ecological studies, identification of dominant ARG classes, and exploratory analyses of environmental AMR reservoirs.	Results are influenced by database selection, sequencing depth, assembly quality, and bioinformatic workflows; environmental assemblies are frequently fragmented.	ARG presence can often be established, but confident reconstruction of ARG–MGE–host linkages may remain uncertain when contigs do not span complete mobile elements or genomic neighborhoods.	(Alcock et al. 2019, Bonin et al. 2023, Daw Elbait et al. 2024, Martiny et al. 2024, Rumi et al. 2024)
Long-read metagenomics (Oxford Nanopore/PacBio)	Improves resolution of genomic architecture by spanning larger DNA fragments, facilitating reconstruction of ARG–MGE–host associations and plasmid structures.	Investigating HGT potential, plasmid-mediated dissemination, mobile resistome dynamics, and identification of potential ARG reservoirs.	Higher sequencing costs and error rates than short-read approaches, although accuracy has improved substantially through chemistry upgrades and hybrid assembly strategies; deep sequencing may still be required for rare taxa and plasmids.	Can substantially strengthen evidence for ARG mobility and host association, but low-abundance elements may remain underrepresented if coverage is insufficient.	(Dai et al. 2022, Mills et al. 2024, Li et al. 2025a)
Stable isotope probing (SIP)	Distinguishes metabolically active micro-organisms from dormant community members by linking isotope incorporation to microbial activity.	Identifying active ARG-carrying populations, evaluating environmentally relevant hosts, and investigating ecological processes associated with resistance dissemination.	Requires isotope incorporation experiments and specialized workflows; activity estimates depend on substrate selection and incubation conditions.	Identifies active populations but does not independently confirm resistance mechanisms, mobility potential, or phenotypic expression of detected genes.	(Li et al. 2022, Hernández et al. 2023)
Functional metagenomics	Provides experimental evidence that recovered DNA fragments confer resistance phenotypes, enabling discovery of ARGs missed by homology-based annotation.	Validation of putative ARGs, identification of novel resistance determinants, and characterization of resistance functions in complex environmental communities.	Dependent on expression in surrogate hosts and selectable screening conditions; some environmentally relevant genes may not be recovered or expressed.	Demonstrates resistance function but does not inherently reveal ecological distribution, host range, or genomic mobility unless integrated with sequencing approaches.	(Murray et al. 2023, Willms et al. 2021)

Despite these strengths, both qPCR and ddPCR have important interpretive limitations for mobile-resistome analysis. As explicitly highlighted in the comparative synthesis table, qPCR/ddPCR quantify elevated ARG abundance but generally cannot determine whether detected genes are plasmid-associated, linked to MGEs, or actively exchanged among hosts without sequencing-based approaches. Consequently, copy-number measurements alone cannot establish genomic context, host identity, or transferability, meaning that these methods quantify resistance burden rather than direct HGT risk (Falcó et al. 2025). In this regard, qPCR is highly effective for targeted surveillance but insufficient when the objective is to determine whether ARGs are integron-linked, plasmid-borne, or embedded within transferable genomic structures. Another major limitation is restricted target coverage, because primer-based assays inherently depend on predefined gene panels and therefore may fail to detect emergent ARG families, rare allelic variants, or compartment-specific resistance determinants lacking primer representation (Yin et al. 2025). The uploaded comparative framework further reinforces that qPCR/ddPCR are fundamentally constrained by targeted assay design and cannot independently resolve genomic mobility or ecological connectivity. Consequently, while qPCR and ddPCR are highly appropriate for sentinel monitoring and hypothesis-driven quantification, they become inadequate for comprehensive resistome discovery or for resolving the ecological architecture of ARG mobility across the water–soil–air continuum.

### Metagenomics and resistome profiling

Shotgun metagenomics provides a broader analytical framework than targeted PCR by enabling simultaneous characterization of ARG diversity, microbial community composition, and co-occurring mobility determinants within the same dataset (Daw Elbait et al. 2024). This systems-level perspective is particularly valuable in environmental surveillance because it allows researchers to identify previously unrecognized resistance reservoirs and to examine co-selection dynamics involving metals, biocides, and virulence factors. Unlike qPCR, metagenomics does not require prior target selection and is therefore substantially more suitable for exploratory resistome discovery and comparative ecological analysis across heterogeneous environments. As outlined in the comparative table (Table 1), short-read shotgun metagenomics is especially advantageous for resistome surveys, identification of dominant ARG classes, and exploratory characterization of environmental AMR reservoirs because it enables comprehensive profiling of ARG diversity within a single analytical workflow. However, the interpretive output of metagenomic workflows remains highly dependent on bioinformatic pipeline design, because database scope, curation strategies, and classification logic vary substantially among commonly used resources and tools, including AMR++/MEGARes and CARD (Alcock et al. 2019, Bonin et al. 2023). These methodological differences can significantly alter ARG annotations, abundance estimates, and inferred resistance profiles, complicating reproducibility and cross-study comparisons (Rumi et al. 2024).

A critical limitation of short-read metagenomics is that fragmented assemblies often fail to resolve ARG–MGE–host linkages, which are central to defining the mobile resistome rather than merely cataloging total ARG abundance (Martiny et al. 2024). The comparative table (Table 1) explicitly emphasizes that ARG presence can frequently be established through short-read metagenomics, yet confident reconstruction of ARG–MGE–host relationships often remains uncertain when contigs fail to span complete mobile elements or genomic neighborhoods. In many environmental datasets, ARG-containing contigs are too short to confidently distinguish chromosomal resistance determinants from plasmid-associated or integron-mediated configurations. Consequently, metagenomics may overestimate ecological risk by identifying ARG presence without confirming transfer potential. This represents one of the clearest situations where metagenomics alone becomes insufficient, particularly when policy-relevant questions require evidence of mobility, dissemination pathways, or host attribution. Methods that analyze flanking regions and local genomic neighborhoods can partially mitigate this issue by identifying mobility-associated signatures such as transposases, insertion sequences, or integron elements (Martiny et al. 2024). Nevertheless, their performance remains constrained in highly fragmented assemblies or strain-complex communities where repeated sequences and genomic heterogeneity impair confident reconstruction. Cross-study comparability also remains problematic because differences in DNA extraction, sequencing depth, library preparation, normalization strategies, and bioinformatic thresholds can generate variability that exceeds underlying biological differences across environmental compartments (Rumi et al. 2024). Therefore, while shotgun metagenomics is indispensable for broad resistome profiling and discovery-oriented studies, it frequently fails to provide definitive evidence of ARG mobility or host linkage without complementary genome-resolved or long-read approaches.

### Long-read sequencing and genome-centric analysis

Long-read metagenomics using platforms such as Oxford Nanopore Technologies and PacBio directly addresses one of the most important limitations of short-read sequencing by spanning ARG–MGE–host architectures within single reads or contiguous assemblies (Dai et al. 2022). This capability is essential when the primary objective is to characterize the mobile resistome rather than the total resistome, because mobility risk depends not only on ARG abundance but also on genomic packaging within transferable elements. Long-read approaches therefore become indispensable when researchers need to determine whether ARGs are plasmid-borne, integron-associated, or linked to high-risk taxa capable of dissemination across environmental interfaces. As summarized in the uploaded comparative framework, long-read metagenomics substantially improves reconstruction of plasmid structures and ARG–MGE–host associations, making it particularly valuable for investigating HGT potential, plasmid-mediated dissemination, and mobile resistome dynamics. In engineered and wastewater systems, long-read sequencing has demonstrated that ARG–MGE associations can shift substantially across treatment compartments even when bulk ARG abundance appears relatively stable, indicating that mobility risk may change independently of total resistome load (Dai et al. 2022). Similarly, in watershed biofilms and natural aquatic systems, long-read metagenomics has resolved reservoir taxa and exchange hubs with greater confidence than short-read assemblies, strengthening ecological inference regarding ARG persistence and transmission pathways (Mills et al. 2024).

From a translational and policy perspective, genome-centric long-read frameworks provide a more biologically meaningful measure of resistome risk because they prioritize transferable ARG configurations rather than simple gene counts (Li et al. 2025a). In this context, long-read sequencing becomes essential whenever surveillance objectives require mechanistic understanding of HGT potential, plasmid dissemination, or reservoir connectivity across the water–soil–air continuum. The comparative table (Table 1) further emphasizes that long-read approaches can substantially strengthen evidence for ARG mobility and host association, thereby overcoming one of the principal inference gaps of short-read metagenomics. However, these advantages are accompanied by important trade-offs. Long-read platforms may exhibit higher per-base error rates than short-read systems, necessitating hybrid correction workflows or stringent filtering to avoid inaccurate ARG classification, particularly when single-nucleotide differences alter functional interpretation among closely related alleles (Dai et al. 2022). Operationally, long-read sequencing also remains more resource-intensive in terms of DNA quality requirements, computational processing, and sequencing cost, which may limit scalability for routine environmental surveillance. Furthermore, uneven recovery of low-abundance plasmids, rare taxa, or fragile organisms can bias mobility inference toward dominant community members unless extraction protocols and sequencing depth are specifically optimized for circular elements and difficult-to-lyse cells (Mills et al. 2024). In addition, the insufficient coverage may still underrepresent rare plasmids or low-abundance mobile elements despite the improved genomic resolution of long-read systems, as described in Table 1. Consequently, long-read metagenomics is not always necessary for broad surveillance applications, but it becomes critical when resolving ARG mobility, plasmid ecology, and host-associated dissemination pathways is the central analytical goal.

### Stable isotope probing (SIP) and functional metagenomics

SIP and functional metagenomics extend resistome analysis beyond compositional profiling by addressing a major unresolved question in environmental AMR surveillance: which organisms are metabolically active and functionally relevant under specific ecological conditions. SIP shifts the analytical focus from “who is present” to “who is active,” enabling direct linkage of ARG carriage to metabolically engaged cells rather than relic or extracellular DNA pools that can inflate apparent resistome size (Li et al. 2022). This distinction is particularly important because DNA-based surveys alone may overestimate ecological and public-health risk by detecting dormant or nonviable organisms incapable of participating in HGT. As emphasized in the uploaded comparative synthesis, SIP is particularly useful for identifying active ARG-carrying populations and for evaluating ecologically relevant hosts involved in resistance dissemination processes. Single-cell SIP coupled with targeted metagenomics has resolved active soil resistomes at high resolution, allowing identification of taxa actively participating in growth-linked ecological processes where ARG exchange is more plausible (Li et al. 2022). Similarly, isotope-labeling approaches in agricultural soils have identified metabolically active antibiotic-resistant populations that differ substantially from the dominant taxa detected through bulk metagenomic sequencing, suggesting that activity-filtered approaches may provide a more risk-relevant representation of environmental AMR dynamics (Hernández et al. 2023).

Functional metagenomics complements SIP by experimentally validating resistance phenotypes rather than relying solely on sequence homology (Murray et al. 2023). This distinction is critical because homology-based pipelines may misclassify distant gene variants or fail to detect entirely novel resistance determinants lacking database representation. As highlighted in the Table 1, functional metagenomics can experimentally demonstrate resistance phenotypes and therefore detect ARGs that remain invisible to conventional homology-based annotation workflows. Functional screening approaches therefore become essential when the goal is to discover emergent ARGs, cryptic resistance mechanisms, or noncanonical determinants associated with stress adaptation, metabolism, or co-selection pressures (Murray et al. 2023). In this regard, functional metagenomics can succeed where conventional metagenomics fails, particularly for identifying previously uncharacterized resistance genes that would remain undetected in reference databases. However, these methods also have substantial ecological and technical constraints. Functional metagenomics depends on successful expression within surrogate laboratory hosts and on experimentally selectable phenotypes, meaning that many environmentally relevant ARGs may remain undetected due to incompatibilities in gene regulation, codon usage, protein folding, or host physiology (Willms et al. 2021). Similarly, SIP approaches are experimentally demanding, require specialized isotopic substrates, and are often limited in throughput and environmental scalability. Importantly, the comparison in Table 1, emphasizes that SIP identifies active microbial populations but does not independently confirm resistance mechanisms or mobility potential, whereas functional metagenomics demonstrates resistance function without inherently resolving ecological distribution, host range, or genomic mobility unless integrated with sequencing approaches. Consequently, SIP and functional metagenomics are not practical replacements for routine surveillance platforms such as qPCR or shotgun sequencing, but they become indispensable when the research objective requires functional validation, activity-resolved ecology, or mechanistic understanding of active resistance dissemination within complex environmental systems.

## ARG dissemination across the water-soil-air continuum

The dissemination of mobile ARGs occurs through a highly interconnected water–soil–air continuum in which environmental compartments function not as isolated reservoirs but as dynamically linked systems that collectively sustain AMR dissemination. Within this continuum, water primarily acts as a transport corridor facilitating ARG movement across ecosystems, soil functions as a long-term reservoir and exchange interface for microbial communities and MGEs, and air serves as an atmospheric dissemination route enabling localized and long-range transport of resistant micro-organisms and extracellular genetic material. Anthropogenic activities including wastewater discharge, agricultural runoff, manure application, aquaculture, urbanization, and industrial emissions intensify these interconnected pathways by introducing antibiotics, resistant bacteria, heavy metals, pesticides, and disinfectants that collectively promote HGT and co-selection. Understanding the environmental resistome through this integrated nexus framework is therefore critical for resolving how ARGs circulate between environmental compartments and ultimately reach human and animal populations.

### ARGs in the water nexus

Aquatic systems constitute major transport corridors within the water–soil–air continuum because they connect anthropogenic sources, environmental reservoirs, and downstream exposure pathways through hydrological movement. Wastewater treatment plants (WWTPs), rivers, groundwater systems, reservoirs, and coastal environments collectively facilitate ARG dissemination by concentrating microbial populations, antibiotics, resistant bacteria, and MGEs under conditions favorable for HGT (Sassi et al. 2025). Municipal, hospital, industrial, and agricultural wastewaters continuously introduce resistant micro-organisms and residual antimicrobials into WWTPs, where dense microbial communities and prolonged bacterial contact enhance conjugative transfer and ARG exchange (Brown et al. 2024, Drane et al. 2024). Although wastewater treatment reduces microbial loads, ARGs and MGEs frequently persist across treatment stages, particularly within activated sludge and biosolids, allowing resistant bacteria and extracellular DNA to enter receiving environments through treated effluents and sludge application (Drane et al. 2024, Aguilar-Rangel et al. 2025, Zhao et al. 2025). Consequently, aquatic systems act simultaneously as reservoirs, amplification hotspots, and dissemination routes for mobile resistomes.

**Figure 1:**
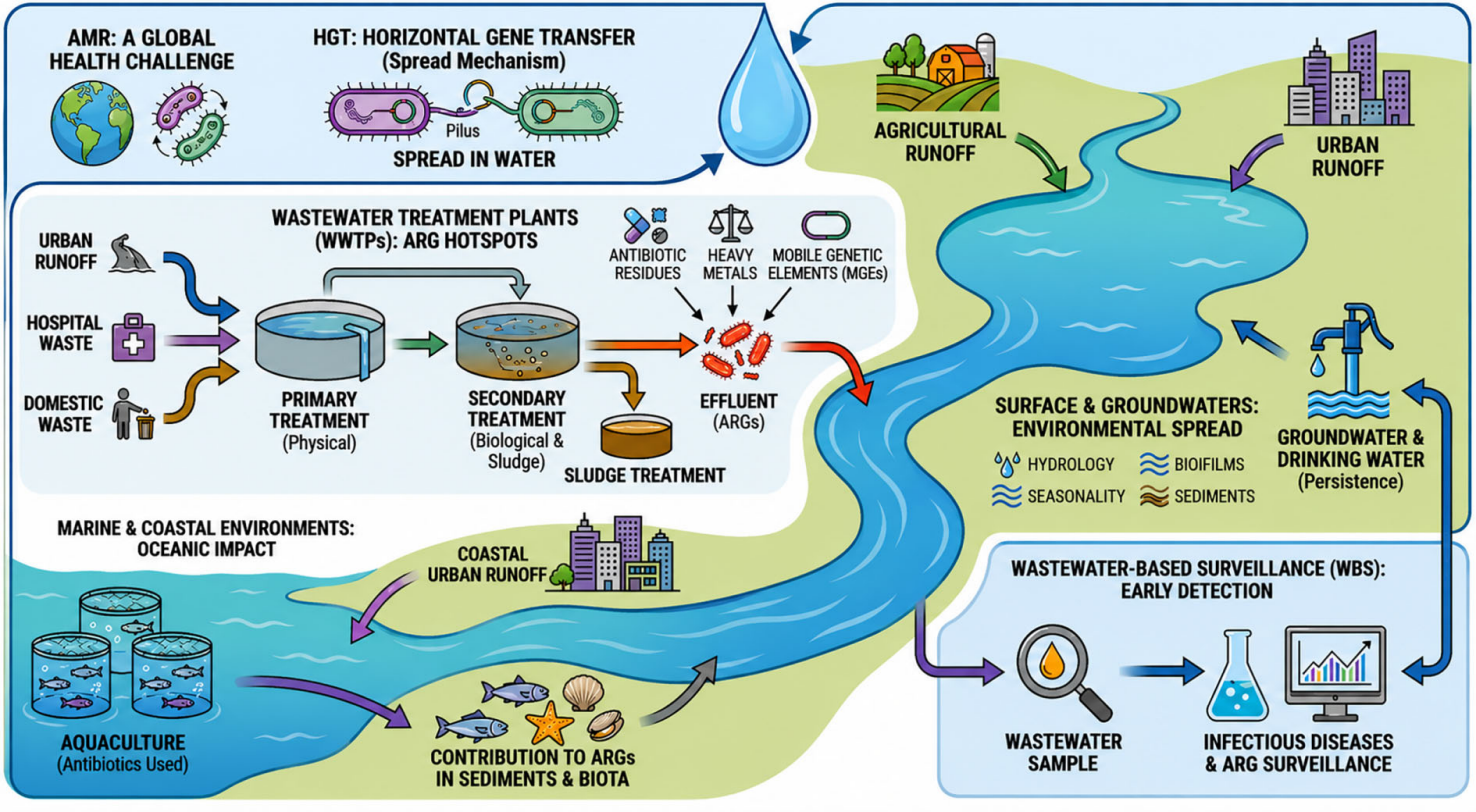
Environmental dissemination of ARGs within the aquatic nexus of the water–soil–air continuum. The figure illustrates the major pathways through which ARGs and MGEs circulate within interconnected aquatic environments. WWTPs receive antibiotic residues, resistant bacteria, and ARGs from domestic, hospital, industrial, agricultural, and urban sources, creating conditions that facilitate HGT. Although wastewater treatment reduces microbial loads, residual ARGs, resistant micro-organisms, and extracellular DNA may persist in treated effluents and biosolids, enabling their release into rivers, groundwater systems, reservoirs, and coastal ecosystems. Hydrological transport, biofilm formation, sediment accumulation, and seasonal environmental processes contribute to the persistence and redistribution of ARGs across aquatic compartments. The figure further highlights the role of wastewater-based surveillance as an emerging tool for monitoring environmental antimicrobial resistance and identifying potential public health risks associated with the dissemination of mobile resistomes.

Hydrological connectivity further enables ARG transport from wastewater systems into rivers, reservoirs, groundwater, and agricultural soils. Surface waters receiving wastewater discharge often contain clinically relevant ARGs associated with Acinetobacter, Pseudomonas, and Enterobacteriaceae, while infiltration through permeable soils facilitates groundwater contamination (Jia et al. 2024, Wang et al. 2024a, Chayña et al. 2025). Seasonal fluctuations, rainfall events, sediment resuspension, and biofilm formation additionally influence ARG persistence and transport within aquatic systems (Barrantes-Jiménez et al. 2025). These processes create continuous feedback between water and soil compartments because contaminated irrigation water introduces ARGs into agricultural soils, while runoff from manure-amended land subsequently returns resistant bacteria and antibiotics to surrounding waterways. In coastal and marine ecosystems, aquaculture operations and urban runoff intensify these dynamics by releasing antibiotics and resistant micro-organisms into estuarine environments, where ARG accumulation in sediments and aquatic biota has become increasingly common (Aa 2025). Collectively, the aquatic compartment functions as the principal circulation pathway linking environmental resistome reservoirs across the broader water–soil–air continuum, as shown in Fig. 1.

### ARGs in the soil nexus

Soil ecosystems function as long-term environmental reservoirs where ARG persistence, microbial interaction, and HGT converge within highly diverse microbial communities. Although antibiotic resistance is evolutionarily ancient and naturally occurs in soil microbiomes (D’Costa et al. 2011), anthropogenic activities including manure application, wastewater irrigation, biosolid deposition, and agricultural intensification substantially amplify the abundance and mobility of environmental ARGs (Qu et al. 2008, Knapp et al. 2010, Durso et al. 2011, Singh et al. 2024, Zeng et al. 2025). Animal manure represents one of the largest contributors to soil resistome enrichment because livestock feces contain abundant tetracycline, sulfonamide, β-lactam, and multidrug resistance genes frequently associated with plasmids, integrons, and transposons (Gu et al. 2020, Peng et al. 2022, Wang et al. 2024b, He et al. 2025, Huang et al. 2025). Once introduced into agricultural soils, these ARGs can persist within microbial communities, interact with indigenous bacteria, and disseminate through HGT under selective pressures imposed by residual antibiotics, heavy metals, and other co-contaminants.

**Figure 2:**
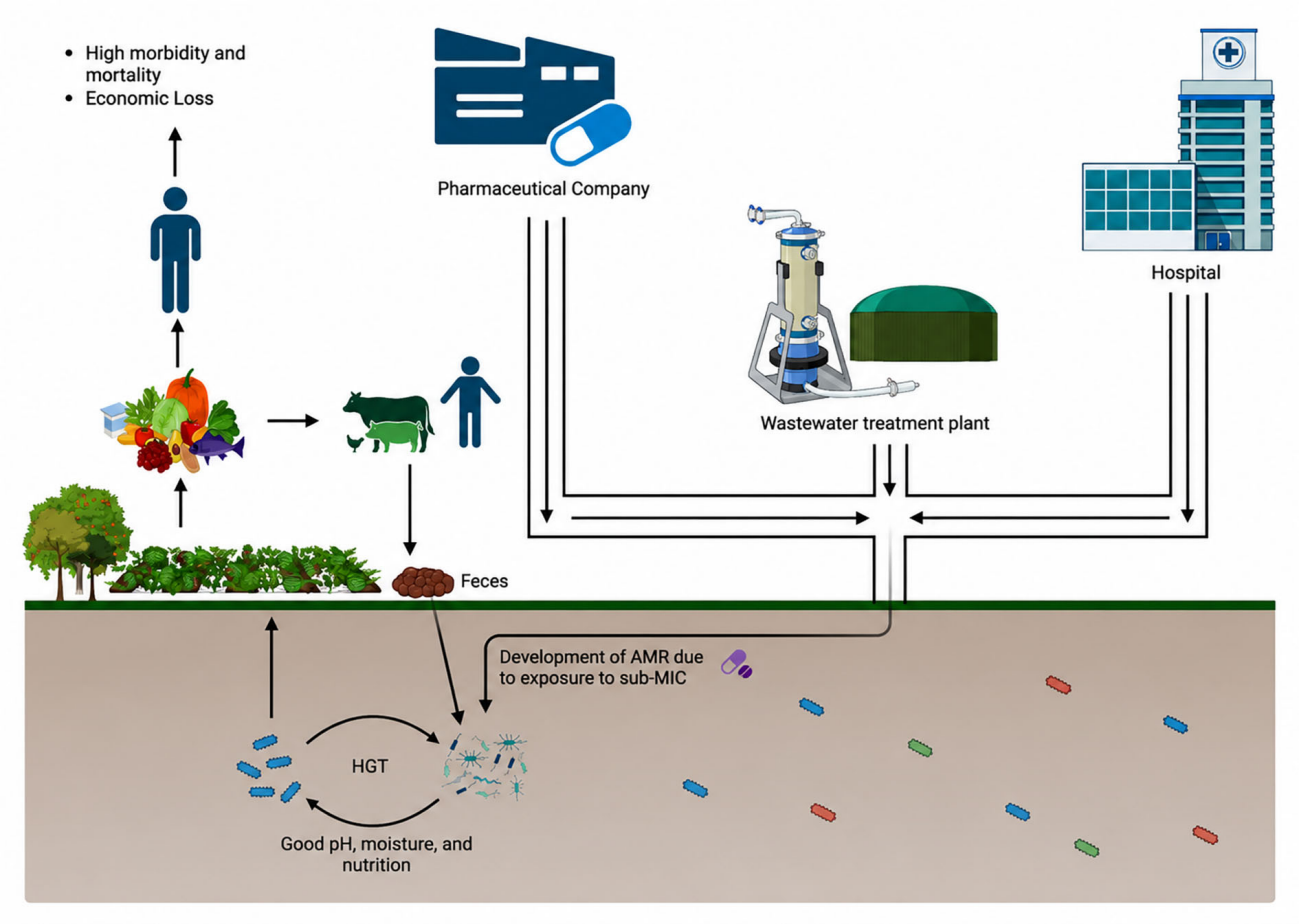
Soil-mediated persistence and horizontal dissemination of ARGs within the water–soil–air nexus. The figure depicts the role of soil ecosystems as major environmental reservoirs and exchange interfaces for ARGs. Resistance determinants originating from pharmaceutical industries, healthcare facilities, WWTPs, livestock production systems, and agricultural activities are introduced into soils through wastewater irrigation, biosolid application, manure amendment, and fecal contamination. Within the soil matrix, favorable environmental conditions, including adequate moisture, nutrient availability, microbial diversity, and biofilm formation, facilitate HGT among indigenous and introduced micro-organisms. Exposure to sub-minimum inhibitory concentrations (sub-MICs) of antibiotics and other environmental contaminants promotes the maintenance, selection, and amplification of mobile ARGs. The figure illustrates how soils function as dynamic ecological interfaces linking human, animal, agricultural, and environmental reservoirs, thereby contributing to the persistence and dissemination of antimicrobial resistance across interconnected ecosystems.

**Figure 3:**
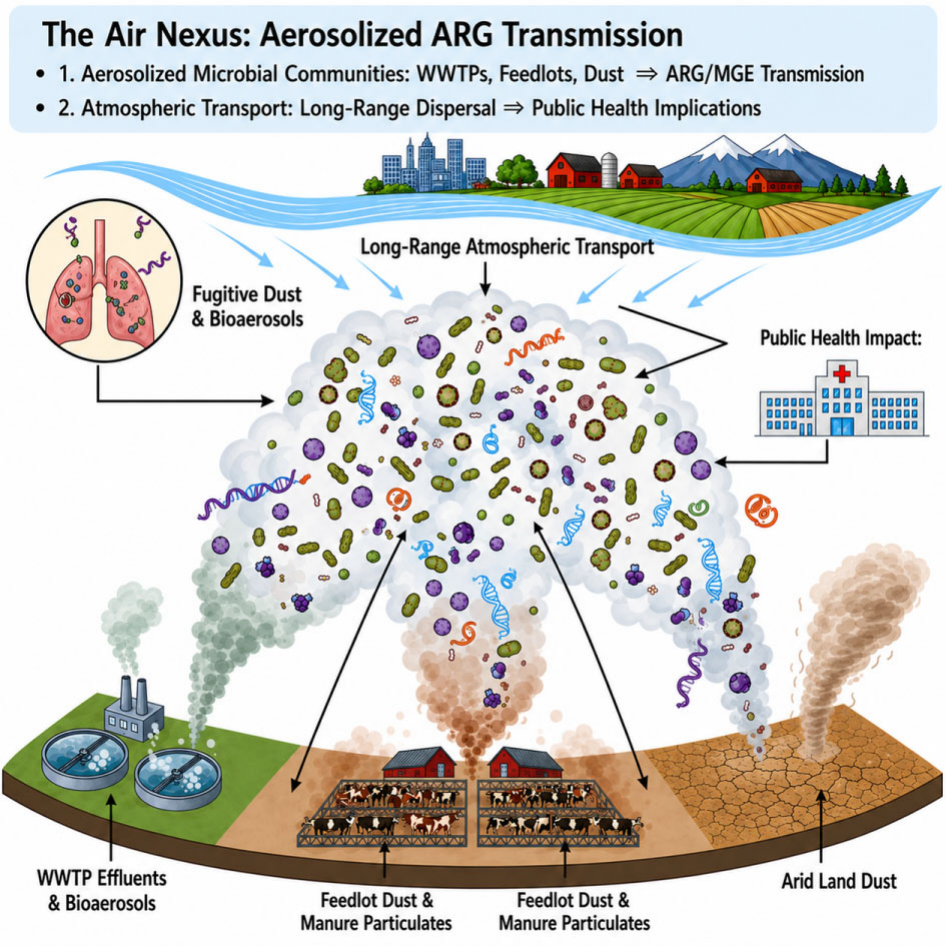
Atmospheric transport and environmental dissemination of ARGs through the air nexus. The figure illustrates the aerosolization, atmospheric transport, and environmental deposition of ARGs, resistant micro-organisms, and MGEs. Bioaerosols generated from WWTPs, concentrated animal feeding operations, livestock facilities, manure particulates, and arid-land dust serve as important sources of airborne resistance determinants. Fine particulate matter and dust particles facilitate the long-range transport of ARG-containing microbial communities across environmental and geographical boundaries. Atmospheric circulation enables the redistribution of resistant micro-organisms through wet and dry deposition processes, reconnecting airborne resistomes with terrestrial and aquatic ecosystems. The figure further highlights potential public health implications associated with inhalation exposure to airborne ARGs and resistant bacteria, emphasizing the atmosphere as a critical dissemination bridge within the integrated water–soil–air continuum and a key component of the One Health framework for understanding environmental antimicrobial resistance.

Within the water–soil–air continuum, soils operate as major exchange interfaces linking aquatic contamination, agricultural practices, and atmospheric dissemination. Wastewater irrigation and biosolid application continuously transfer ARGs from WWTPs into terrestrial environments, while rainfall runoff and soil erosion mobilize resistant bacteria back into rivers, reservoirs, and groundwater systems (Lüneberg et al. 2018, Ye et al. 2021, Błażejewska et al. 2022, Zhu et al. 2022). The rhizosphere represents a particularly active hotspot for ARG exchange because root exudates support dense microbial populations and close cellular interactions that facilitate HGT (Vellend 2010, Rankin et al. 2011, Soucy et al. 2015, Booth et al. 2016, Van De Guchte 2017, Zhu et al. 2019, Brunel et al. 2020). ARGs associated with MGEs frequently accumulate in rhizosphere microbiomes, increasing their dissemination potential across phylogenetically diverse taxa. Soil particles and manure-derived dust can also become aerosolized during agricultural activities, thereby linking terrestrial resistomes to atmospheric transport pathways. Consequently, soils function not merely as passive reservoirs but as highly active ecological interfaces where ARG persistence, amplification, and cross-compartment dissemination continuously occur within the interconnected water–soil–air nexus, as shown in Fig. 2 and Fig. 3.

### ARGs in the air nexus

The atmosphere represents the most mobile compartment within the water–soil–air continuum because aerosolized resistant bacteria, extracellular DNA, and MGEs can undergo localized and long-range transport across environmental and geographical boundaries. Bioaerosols generated from WWTPs, concentrated animal feeding operations (CAFOs), composting facilities, agricultural soils, and urban environments contain diverse ARGs and MGEs, including tetracycline, sulfonamide, β-lactam, and multidrug resistance determinants frequently associated with Class 1 integrons (Yang et al. 2018, Chen et al. 2022, Gwenzi et al. 2022, Lee and Yoo 2022, Habibi et al. 2024, Ding et al. 2025). In WWTPs, aeration processes, sludge handling, and mechanical turbulence facilitate aerosol generation, whereas manure spreading, livestock activity, tillage, and wind erosion release ARG-laden dust particles from agricultural systems into the atmosphere (Zhao et al. 2022, Zhao et al. 2024, Texas Tech University 2015). Atmospheric transport is strongly influenced by particle size and meteorological conditions: coarse particles (>5 µm) generally settle near their source, whereas fine particles (<2.5 µm) can remain suspended for prolonged periods and travel across regional and intercontinental air currents (McEachran et al. 2015, Yan et al. 2021). Fine particulates may adsorb extracellular DNA, antibiotics, heavy metals, and organic matter, creating protective microenvironments that sustain microbial viability and potentially facilitate HGT during transport (Gillings et al. 2015, Khan et al. 2024).

Long-range atmospheric deposition further connects geographically distant resistomes through dust storms, biomass burning events, and transboundary aerosol circulation that redistribute ARGs and resistant micro-organisms across terrestrial and aquatic ecosystems (Chen et al. 2024, Fan et al. 2025, Kormos et al. 2025). Saharan dust transport, for example, has been associated with the intercontinental dispersal of viable microbial communities and resistance-associated genetic material into distant environments with limited direct antibiotic exposure (Fan et al. 2025, Kormos et al. 2025). Wet and dry deposition subsequently transfer airborne ARGs back into soils, rivers, agricultural surfaces, and coastal waters, effectively reseeding environmental reservoirs and reinforcing cross-compartment recirculation within the nexus (Perfumo and Marchant 2010, Wang et al. 2021). The detection of clinically relevant ARGs in PM2.5 and PM10 from hospitals, urban air, and livestock environments highlights the growing public health relevance of airborne resistomes because inhalation provides a direct exposure pathway through which ARGs may interact with respiratory microbiomes and opportunistic pathogens (Zhao et al. 2021, Chen et al. 2024, Khan et al. 2024). Within the integrated water–soil–air framework, the atmosphere therefore functions not only as a transport route but also as a dynamic dissemination bridge that drives aerosolization, long-range redistribution, environmental deposition, and large-scale circulation of mobile resistomes.

The table summarizes the primary sources of ARGs, key environmental transport routes, and dominant ARG classes and MGEs within air, soil, and water systems (Table 2). Airborne ARGs are largely associated with bioaerosols from anthropogenic point sources, soil resistomes are shaped by agricultural inputs and rhizosphere-driven microbial interactions, and aquatic environments act as major convergence zones for diverse ARG inputs from wastewater, agricultural runoff, and industrial discharges. HGT mediated by plasmids, transposons, and integrons represents a shared mechanism underpinning ARG dissemination across all compartments, highlighting the interconnected nature of environmental resistomes within a One Health framework.

**Table 2:** Sources, transport pathways, and dominant ARGs and MGEs across environmental compartments (Water–Soil–Air continuum).

Category	Air	Soil	Water	References
**Sources of ARGs**	Airborne ARGs are primarily associated with bioaerosols originating from animal husbandry, healthcare facilities, and WWTPs. Pig farms exhibit particularly high airborne ARG burdens.	Soil resistomes are largely shaped by agricultural inputs, including irrigation with untreated wastewater, which introduces antibiotics, resistant bacteria, and extracellular resistance genes. The rhizosphere is a major hotspot for ARG exchange and enrichment.	Aquatic environments accumulate ARGs from municipal and hospital effluents, industrial discharges, agricultural runoff, and untreated sewage. Seawater typically harbours a broader ARG repertoire than freshwater systems.	(Grohmann 2013, Lüneberg et al. 2018, Chen et al. 2022, Kour et al. 2023, Lu et al. 2024, Guo et al. 2025, Jiang et al. 2025, Zulkifle et al. 2025)
**Transport pathways**	ARG dissemination occurs via particulate matter (PM₂.₅ and PM₁₀) and bioaerosols, with atmospheric processes such as wind dispersion and precipitation facilitating deposition into terrestrial and aquatic environments.	Hydrological flow pathways promote ARG accumulation and redistribution, particularly for mobile antibiotics such as sulfamethoxazole. HGT is a central mechanism driving ARG propagation.	ARG spread is mediated by HGT (conjugation, transformation, and transduction). WWTPs act as major hotspots for ARG exchange.	(Dodd 2012, Grohmann 2013, Lüneberg et al. 2018, Wang et al. 2021, Chen et al. 2022, Kour et al. 2023, Lu et al. 2024, Wang et al. 2024a)
**Dominant ARGs and MGEs**	Tetracycline, sulfonamide, and β-lactam resistance genes dominate. Transposons and integrative elements facilitate ARG mobility in bioaerosols.	Multidrug resistance and vancomycin resistance genes are prevalent. Plasmids, transposons, and integrative conjugative elements (ICEs) drive ARG dissemination.	β-lactam resistance genes (e.g. blaTEM, blaCTX-M), sulfonamide, and tetracycline resistance genes dominate. Integrons and transposases (e.g. tnpA) are key vectors.	(Grohmann 2013, Lee and Yoo 2022, Zhou et al. 2023, Lu et al. 2024, Guo et al. 2025, Jiang et al. 2025, Sharma et al. 2025, Zulkifle et al. 2025)

## The critical role of co-selection

Exposure to non-antibiotic stressors, particularly heavy metals and antibacterial biocides, exerts a potent co-selective pressure that maintains and propagates ARGs in environmental matrices, primarily through their genetic linkage on MGEs. Compelling evidence for this co-selection spans diverse environmental compartments. Dickinson et al. provide critical historical validation using a sediment core archive, showing that zinc pollution from early 20th-century industry selected for bacterial resistance to antibiotics like cefotaxime decades before their clinical use. The strong correlation between zinc concentration, antibiotic resistance in isolates, and the anthropogenic integron marker *intI1* strongly implies MGE-mediated genetic linkage (Dickinson et al. 2019). While this palaeontological approach powerfully isolates metal-driven selection from antibiotic pressure, its correlative nature cannot definitively map the physical co-localization of metal and antibiotic resistance genes on specific MGEs.

Controlled experimental studies address this mechanistic gap. One study demonstrates that co-exposure to the organoarsenical roxarsone (a feed additive) and heavy metals (Zn/Cu) synergistically selects for multidrug-resistant *E. coli* mutants. The observed upregulation of multidrug efflux pumps (*acrB, mdtF*) and concurrent resistance to metals and antibiotics points strongly toward co-resistance mechanisms, likely plasmid-encoded (Liu et al. 2026). This study powerfully links phenotype to genotype but uses a model strain in a simplified system, leaving open questions about the prevalence and dynamics of these linkages in complex microbial communities. To translate findings into actionable risk thresholds, Arya et al. developed a predictive mathematical model, proposing Minimal Co-Selective Concentrations (MCSCs) for metals. Their model indicates that copper (5.5 mg/l) and zinc (1.6 mg/l) can co-select for linked ARGs at concentrations far below their toxicity thresholds and often under regulatory limits (Arya et al. 2021). This theoretical framework is invaluable for risk assessment but requires validation with empirical data on specific, co-localized MRG-ARG pairs in real-world samples.

Finally, Ting Zhong et al. extend the co-selection paradigm beyond water and soil to the atmosphere. Their finding that atmospheric ARG abundance correlates more strongly with biocide and metal resistance genes (BRGs, MRGs) than with antibiotics themselves underscores the ubiquity of this pressure. They posit that co-selection among these resistance derivatives ensures the stable persistence of ARGs in air (Zhang et al. 2025a). This highlights a significant research frontier: identifying the specific MGEs (e.g. plasmids, cassettes in integrons) that physically carry these linked resistance genes in airborne bacteria. Sub-inhibitory concentrations (SICs) of antibiotics, pervasive in environmental matrices due to incomplete degradation and dilution, exert profound selective pressures that critically amplify the dissemination of AMR by simultaneously increasing HGT frequency and mutation rates (Ding et al. 2022, Ismail et al. 2025).

A primary mechanism is the induction of conjugative transfer. SICs of diverse antibiotic classes, including carbapenems, fluoroquinolones, cephalosporins, and aminoglycosides, significantly increase plasmid conjugation frequency between clinically relevant pathogens (Ding et al. 2022). This promotion is mechanistically linked to the upregulation of genes encoding the Type IV Secretion System (T4SS) and plasmid transfer (*tra*) operons in the donor bacterium (Ding et al. 2022). The effect is not confined to conjugation. SICs of at least twelve antibiotics from various classes also promote cell-to-cell plasmid transformation within *E. coli* biofilms, a process further enhanced under the anaerobic conditions typical of the gut and soil micropores (Hirayama et al. 2025). Concurrently, SICs elevate the genetic raw material for selection by increasing mutation rates. Exposure to SICs of antibiotics like rifampin and vancomycin increases the mutation rate to streptomycin resistance in *Staphylococcus aureus* by approximately an order of magnitude, a response dependent on the RecA-mediated SOS stress system (Ismail et al. 2025). This creates a dual-risk scenario: SICs enhance the transfer of existing resistance genes while also accelerating the *de novo* generation of resistance mutations. These studies demonstrate that SICs act as broad-spectrum enablers of AMR evolution. Methodologically, they employ models from *in vitro* assays to *in vivo* larvae and biofilm systems, strengthening ecological relevance (Ding et al. 2022, Hirayama et al. 2025). A key consensus is the generality of the effect across antibiotic classes. A significant gap remains in quantifying these phenomena in complex environmental settings under realistic, fluctuating antibiotic gradients. Future research must prioritize *in situ* measurements to model the contribution of SICs to resistome dynamics in the water-soil-air nexus accurately.

## Risk assessment and mitigation strategies

Environmental antibiotic resistance represents a measurable public health risk that requires formal risk assessment and targeted mitigation (Larsson and Flach 2022, Zhang et al. 2025b). The environmental dissemination of mobile ARGs through water, soil, and air creates exposure pathways that extend beyond clinical settings and challenge conventional surveillance systems (Uluseker et al. 2021, Larsson and Flach 2022, Chi et al. 2025, Sassi et al. 2025). Quantitative models are integral in assessing the probability, magnitude, and uncertainty of human and ecological exposure to mobile ARGs, enabling estimation of transmission dynamics, persistence, and population-level risk across environmental compartments (Larsson and Flach 2022, Klümper et al. 2025, Sassi et al. 2025). These environmental transmission dynamics underscore the need to move beyond descriptive mapping of antimicrobial resistance toward targeted intervention strategies capable of limiting the spread and persistence of mobile resistance genes across environmental systems (Larsson and Flach 2022, Klümper et al. 2025).

### Quantitative risk assessment models

Quantitative risk assessment models are increasingly used to translate environmental ARG occurrence into estimates of health-relevant risk (Klümper et al. 2025). One approach adapts Quantitative Microbial Risk Assessment (QMRA) frameworks to antibiotic resistance, focusing on ingestion or contact exposure to ARGs rather than to specific pathogens (Zhang et al. 2022, Klümper et al. 2025, Shin et al. 2026). For example, a 2024 study applied QMRA to private well water contaminated with ARGs, estimating the daily probability that residents ingest at least one resistance gene and the size of the exposed population (Burch et al. 2024). The authors estimated that hundreds to thousands of individuals could ingest genes such as *intI1* and *tet(X)* daily from untreated wells, particularly in regions affected by livestock waste, highlighting rural groundwater as an underrecognized exposure pathway (Burch et al. 2024). However, a central limitation remains that detection of ARG DNA does not necessarily imply viable resistant pathogens or infection risk (Larsson and Flach 2022, Zhang et al. 2022, Burch et al. 2024). Current models can quantify exposure to genetic material, but linking ARG intake to clinically meaningful outcomes require further data on HGT rates and dose–response relationships for resistance determinants (Smalla et al. 2018, La Rosa et al. 2025).

Beyond direct exposure modeling, several frameworks have been developed to rank the relative risk posed by ARGs across environments (Larsson and Flach 2022, Zhang et al. 2022). These methods incorporate gene abundance, association with MGEs, host range, and presence in human pathogens to estimate mobilization and clinical relevance (Zhang et al. 2022). Tools such as MetaCompare project metagenomic samples into a hazard space and generate resistome risk scores, consistently identifying higher risk in hospital wastewater compared with agricultural or treated effluents (Oh et al. 2018, Zhang et al. 2025a). At a global scale, analysis of more than 4500 metagenomes classified approximately one-quarter of detected ARGs as high risk, particularly multidrug resistance genes with broad host range, and machine-learning models predicted resistome hotspots with over 75% accuracy (Zhang et al. 2022). A consistent finding across studies is that mobility and pathogenic host association are more informative than ARG abundance alone when evaluating public health risk (Raza et al. 2022, Shin et al. 2026).

These models represent meaningful progress toward a One Health framework for environmental AMR risk assessment (Oliveira et al. 2024, La Rosa et al. 2025). They enable comparisons across sites, prioritize surveillance targets, and support scenario analyses evaluating potential risk reduction from interventions (Klümper et al. 2025). For instance, a transmission-chain risk analysis of hospital wastewater demonstrated that although treatment removed over 90% of antibiotics, high-risk ARGs persisted, with more than 80% of ARGs in receiving waters classified as high risk (Chi et al. 2025). The study further showed that ARG dissemination risk was substantially higher in water than in sediments, underscoring the importance of aquatic pathways (Chi et al. 2025). Despite these advances, key limitations remain, including uncertainty in health outcome translation, limited incorporation of in situ HGT dynamics, and lack of standardized risk metrics across studies. Addressing these gaps is critical for improving predictive reliability and informing mitigation targets (Smalla et al. 2018).

### Intervention strategies

Reducing environmental AMR requires interventions at multiple points along the transmission chain (Smalla et al. 2018, Zhang et al. 2025a). Conventional WWTPs are not designed to remove ARGs, and treated effluents frequently contain resistant bacteria and genes at levels exceeding those of receiving waters (Oh et al. 2018, Uluseker et al. 2021, Zhang et al. 2025a). Advanced treatment technologies have therefore been explored to reduce environmental loading (Raza et al. 2022). Membrane bioreactors consistently outperform conventional activated sludge, achieving up to 1–7 log reductions in key ARGs due to enhanced microbial and particle retention (Li et al. 2019, Pazda et al. 2019, Zhan et al. 2019). Advanced oxidation processes, particularly ozonation, effectively inactivate resistant bacteria and reduce ARG copy numbers in hospital wastewater, with >99.9% removal of culturable resistant organisms reported in some systems (Baghal Asghari et al. 2021, La Rosa et al. 2025). However, UV disinfection alone often fails to sufficiently degrade extracellular DNA, raising concerns about downstream gene uptake. Multi-barrier treatment combining biological, physical, and chemical processes is increasingly viewed as necessary, though cost, energy demand, and by-product formation remain important constraints (Smalla et al. 2018, Uluseker et al. 2021).

Biological strategies such as phage therapy are also being explored as targeted approaches to reduce antibiotic-resistant bacteria in environmental systems. Lytic bacteriophages can selectively eliminate resistant strains without broadly disrupting microbial communities (Gutiérrez et al. 2016). Experimental studies demonstrate that phage application in wastewater systems can significantly reduce multidrug-resistant bacteria and shift microbial community composition away from pathogenic taxa (Gutiérrez et al. 2016, Pazda et al. 2019, Pallavali et al. 2023). Nonetheless, challenges include phage delivery, potential bacterial resistance to phages, risks of transduction, and regulatory uncertainty, and at present, phage-based interventions remain complementary rather than standalone solutions (Smalla et al. 2018).

Upstream source control is widely recognized as essential. Pharmaceutical manufacturing, hospitals, and intensive agriculture contribute disproportionately to environmental AMR loading (Tang et al. 2017, Rayan 2023). In response, the WHO released guidance in 2023 calling for stricter limits on antibiotic discharges from manufacturing facilities (Rayan 2023). Agricultural controls, including restrictions on non-therapeutic antibiotic use and improved manure management, further reduce environmental inputs (Tang et al. 2017). While implementation varies by region, integrating source control with downstream treatment and surveillance offers the most sustainable approach (Smalla et al. 2018, Zhang et al. 2025a). In combination, quantitative risk assessment and multi-level interventions provide a framework for mitigating the environmental dimensions of antimicrobial resistance within a One Health paradigm.

## Recommendations for future research

Future environmental AMR research must prioritise three interconnected goals: methodological harmonisation, risk–differentiated analysis, and mechanistic *in situ* validation. First, standardise methodologies for mobile resistome surveillance. Short–read sequencing alone is inadequate for accurately linking resistance genes to plasmids and MGEs, while long–read sequencing, despite its advantages, is prone to errors and may fail to capture small plasmids. Hybrid sequencing approaches that integrate short– and long–read data overcome these limitations by enabling more accurate reconstruction of mobile genomic contexts. Consequently, hybrid assemblies provide a more reliable framework for resolving AMR gene–MGE associations and should be prioritised in future research to improve the accuracy and completeness of resistome analyses (Juraschek et al. 2021). Second, move from descriptive ARG cataloguing to predictive, risk–ranked frameworks. Tools such as MetaCompare 2.0, which calculate separate Ecological and Human Health Resistance Risk scores, exemplify the shift needed to prioritise genes with high mobility and clinical relevance (Rumi et al. 2024). Future efforts should expand these frameworks to incorporate quantitative HGT rates and to validate risk predictions against real–world clinical outcomes. Third, quantify HGT rates under realistic environmental conditions using SIP. This is a critical knowledge gap that current genomic surveys alone cannot fill. We recommend combining SIP with long–read metagenomics to directly measure *in situ* HGT frequencies. Specifically: SIP identifies metabolically active ARG hosts that are engaged in growth–linked processes—exactly the conditions where HGT is most plausible. Long–read sequencing resolves whether the ARGs carried by those active hosts are located on MGEs (plasmids, integrons, transposons). Together, this pairing moves beyond presence/absence to actual transfer frequency estimates under environmentally relevant conditions (e.g. antibiotic gradients, nutrient fluctuations, and interspecies competition). Pilot studies in wastewater biofilms, manure–amended soils, and aerosol microenvironments are urgently needed to benchmark these approaches. Fourth, integrate multi–compartment surveillance across the water–soil–air nexus. Current monitoring priorities have shifted from simple ARG detection toward understanding the ecological and mechanistic processes that govern resistome mobility and persistence across environmental compartments. Contemporary surveillance frameworks increasingly integrate metagenomics, long–read sequencing, eDNA, and quantitative molecular approaches to resolve ARG–host associations, characterise MGEs, and identify hotspots of HGT in wastewater systems, agricultural soils, biofilms, aerosols, and urban ecosystems (Mourão et al. 2025, Pugazhendhi et al. 2026). Particular emphasis should be placed on tracking clinically relevant ARGs linked to plasmids, integrons, transposons, and bacteriophages, which facilitate dissemination across taxonomic and environmental boundaries. Fifth, prioritise interventions that reduce co–selection pressures. Intervention priorities should focus on reducing selective pressures generated by antibiotic residues, heavy metals, biocides, and microplastics, while enhancing wastewater treatment efficiency and implementing integrated One Health surveillance networks. Such measures are increasingly recognised as essential for disrupting environmental transmission pathways and mitigating the emergence and spread of mobile resistomes across interconnected ecosystems (Meradji et al. 2025, Sassi et al. 2025, Hossain et al. 2026, Zhou et al. 2023).

## Conclusions

The mobile resistome across the water–soil–air continuum is sustained by HGT via MGEs and amplified by anthropogenic co–selection pressures. Rather than restating the detailed mechanisms and methodological comparisons already covered, we conclude by identifying three priorities for environmental AMR surveillance and intervention. First, surveillance must shift from ARG abundance to mobility potential. Current risk assessments that rely on gene copy numbers without genomic context overestimate risk where ARGs are chromosomally embedded and underestimate risk where low–abundance ARGs reside on highly transferable plasmids. Integrating long–read sequencing and functional metagenomics into routine monitoring is essential to resolve ARG–MGE–host linkages. Second, interventions must target transmission hotspots across compartments. WWTPs, manure–amended soils, and concentrated animal feeding operations are not passive receptors but active reactors that amplify ARG diversity and HGT. Upstream source control (e.g. antibiotic discharge limits from pharmaceutical manufacturing, restricted non–therapeutic agricultural use) combined with downstream barriers (membrane bioreactors, ozonation, composting optimization) offers the most effective mitigation. Third, methodological standardization is urgently needed. Without harmonized protocols for sampling, DNA extraction, sequencing depth, and bioinformatic analysis, cross–study comparisons will remain unreliable, and risk assessment models will lack robust input data. Adoption of minimum reporting standards (e.g. MIxS) and interlaboratory calibration studies should be prioritized. Addressing environmental AMR requires treating the water–soil–air continuum as an integrated system. The tools exist; what is needed now is coordinated global action within a One Health framework.

## Data Availability

Not applicable; no new datasets were generated or analyzed.
